# Early and Genetic Biomarkers in Alzheimer's Disease: Role of Metabolism and Neuroinflammation in the Cognition of Coastal Peruvians

**DOI:** 10.1002/alz.092291

**Published:** 2025-01-09

**Authors:** Jonathan Adrian Zegarra‐Valdivia, Harold Alessandro Arana‐Nombera, Leandro Aron Perez‐Fernandez, Milagros del Rocio Casimiro‐Arana, Reyna Elizabeth Alamo‐Medina, Viviana Nayelli Gallegos‐Manayay, María del Rosario Oliva‐Piscoya, Eduardo Sebastián Abanto‐Saldaña, Nobuko Vásquez‐Zuñe, Maria Celinda Cruz Ordinola, Carmen Paredes Manrique

**Affiliations:** ^1^ Universidad Señor de Sipán, Chiclayo Peru; ^2^ Universidad San Martín de Porres, Chiclayo Peru; ^3^ Universidad Tecnológica del Perú, Arequipa Peru

## Abstract

**Background:**

Alzheimer's Disease (AD) has been associated with neurocognitive, metabolic, and neuroinflammatory alterations. Currently, there are no useful biomarkers in low‐resource countries, and genetic risk/protection factors in Peruvians are unknown.

Objective: To establish the first serum bank in the northern part of the country for the analysis of participants with mild cognitive impairment (MCI) or AD. This will enable the identification of a profile of risk/protection factors in the Peruvian population. We will also analyze the interaction between levels of neurocognition, IGF‐I, and neuroinflammation as potential biomarkers for early detection of cerebral deterioration.

**Method:**

This is a quantitative, cross‐sectional, retrospective case‐control study. We will identify the neurocognitive profile and serum biomarkers (IGF‐I, TNF‐α, interleukins) of adult participants (>50 years old), both healthy individuals and those previously identified and diagnosed with MCI or AD by a specialist (neurologist, geriatrician, or psychiatrist).

**Results:**

This is an ongoing project; to date, we have assessed over 100 participants aged 50 and above. We are in the process of evaluating both control subjects and patients with Mild Cognitive Impairment (MCI) and Alzheimer's Disease (AD). We aim to continue our assessments and complete blood sample collections throughout the year.

**Conclusion:**

This study proposes to create the first serum bank for the study of genetic and non‐genetic biomarkers associated with dementia in the northern part of the country. We will use the triad of IGF‐1, neuroinflammation, and neurocognition biomarkers for early diagnosis in MCI and AD. Additionally, this will enable new genetic studies to identify risk/protection factors in Peruvians.

